# Reduced fertility from better access to contraception may not improve women’s health

**DOI:** 10.1073/pnas.2110170118

**Published:** 2021-07-16

**Authors:** David Lam

**Affiliations:** ^a^Department of Economics, University of Michigan, Ann Arbor, MI 48104;; ^b^Institute for Social Research, University of Michigan, Ann Arbor, MI 48104

Fertility rates have declined dramatically in most countries in Asia and Latin America in the last 50 y, with slower declines in sub-Saharan Africa (1, 2). For example, the total fertility rate (TFR), the number of children a woman would have over her lifetime if she experienced the age-specific fertility rates observed in a given year, fell from around 6.0 or higher to around or below the replacement rate of 2.1 between 1960 and 2019 in all of the nine large Asian and Latin American countries shown in Table 1 (1). In Bangladesh, the focus of this Commentary, the TFR fell from 6.9 in 1970 to 2.0 in 2019, a remarkable decline that few would have thought possible in the 1960s. As seen in Table 1, when fertility began to decline in these countries, usually in the 1970s, it fell at a rapid rate. Many factors contributed to these rapid fertility declines. Couples offset declines in infant and child mortality by having fewer births; access to family planning increased, allowing women to better choose the number and timing of births; rapid social and economic change, including urbanization and increases in women’s education, motivated parents to have fewer children and invest more in the health and education of those children (2).

**Table 1.** Total fertility rate for selected Asian and Latin American countries, 1960 to 2019 (United Nations Population Division estimates)

Country	1960	1965	1970	1975	1980	1985	1990	1995	2000	2005	2010	2015	2019
Bangladesh	6.7	6.9	6.9	6.8	6.4	5.5	4.5	3.7	3.2	2.7	2.3	2.1	2.0
Brazil	6.1	5.7	5.0	4.4	4.0	3.5	2.9	2.6	2.3	2.0	1.8	1.8	1.7
China	5.8	6.4	5.7	3.9	2.6	2.7	2.3	1.7	1.6	1.6	1.6	1.7	1.7
Colombia	6.7	6.3	5.3	4.4	3.9	3.3	3.1	2.9	2.6	2.3	2.0	1.9	1.8
India	5.9	5.8	5.6	5.2	4.8	4.5	4.0	3.7	3.3	3.0	2.6	2.3	2.2
Indonesia	5.7	5.6	5.5	5.0	4.4	3.7	3.1	2.7	2.5	2.5	2.5	2.4	2.3
Mexico	6.8	6.8	6.6	5.9	4.8	4.0	3.5	3.0	2.7	2.5	2.3	2.2	2.1
Thailand	6.1	6.1	5.6	4.5	3.4	2.6	2.1	1.9	1.7	1.6	1.5	1.5	1.5
Vietnam	6.3	6.5	6.5	6.0	5.0	4.2	3.6	2.7	2.0	1.9	1.9	2.0	2.1

Now that we are decades away from the early stages of rapid fertility decline in these countries, it is interesting to look at the life trajectories of the women and children who were affected by the introduction of family planning programs. The PNAS paper by Barham et al. (3) allows us to do that, providing an intriguing analysis of the impact of a well-known family planning program in Bangladesh on the later life health and well-being of the women who were participants in the program. The surprising conclusion of this carefully executed study is that women who were in the treatment area do not have measurably better health than women in the comparison area 35 y later, despite the fact that they had fewer children, longer child spacing, and younger age of completed childbearing. If anything, the women in the treatment area had moderately worse health than women in the comparison area. The authors also do not find measurable improvements in economic outcomes for women in the treatment area.

The Barham et al. study takes advantage of unusually rich data to look at these important questions. The Matlab maternal and child health/family planning program (MCH/FP), introduced in the Matlab subdistrict of Bangladesh in 1977, provided some of the earliest evidence that access to family planning could lead to increased contraceptive use and reduced fertility in a low-income rural setting (4, 5). The Matlab MCH/FP is one of the best known and most widely studied family planning interventions in the world. A large literature has provided compelling evidence that the program increased contraceptive use and lowered fertility in the treatment area (6, 7). Research also indicates that these fertility declines were associated with improvements in children’s outcomes, including health, education, and cognitive function (7–10).

While the MCH/FP was not a randomized controlled trial in the standard sense, it had a quasi-experimental design, which consisted of a geographically contiguous treatment area and a comparison area that included two areas adjacent to the treatment area (3). Both the treatment and comparison areas were part of an existing Demographic Surveillance System (originally created for evaluating cholera vaccines), providing an excellent platform for implementing a study of family planning and health interventions. As numerous papers using the Matlab data have shown, including Barham et al. (3), the treatment area looked very similar at baseline to the comparison area on key social, demographic, and economic characteristics. This provides the basis for a large body of research built around analyzing the impact of the MCH/FP intervention on a variety of outcomes.

Continued follow-up of households in the treatment and control areas has allowed researchers to look at the long-run impacts of the program. The research team for the Barham et al. study includes researchers who have been involved in analyzing the Matlab MCH/FP since its inception, and who developed a series of well-designed follow-up surveys of the households in the treatment and comparison areas.

Barham et al. show that women who were childbearing age in the treatment area when the MCH/FP began ended up with 0.5 to 0.7 fewer births than women in the comparison area, depending on their age when the program began, the result of significantly higher levels of contraceptive use. In addition to having fewer children, women in the treatment area tended to have longer birth intervals and younger age at last birth. As the authors point out, there are many reasons to think that these changes would lead to better health outcomes for these women in later life. Surprisingly, however, the estimated MCH/FP treatment effects on later adult health imply that women in the treatment area at the time of the program had 0.07 SD worse overall health 35 y later (*P* < 0.05), driven by 0.12 SD worse health in the respiratory domain (*P* < 0.05) and 0.05 SD worse health in the metabolic domain (*P* = 0.138).

The obvious question is why lower fertility, longer child spacing, and an earlier end of childbearing did not lead to better health for women in later life. Barham et al. provide one possible explanation in their paper. The authors find that the prevalence of being overweight increased in the treatment group for all three of the cohorts analyzed. In their analysis of mediating factors, they find that controlling for body mass index (BMI) explains about one-third of the poorer metabolic component for the women in the MCH/FP treatment group. While part of the increase in BMI involves a reduction in the proportion of women who are underweight, the upward shift of the BMI distribution also leads to an increase in the percentage who are overweight. The nutrition transition that was seen in much of Bangladesh over this period appears to have been somewhat exaggerated in the MCH/FP treatment area, potentially offsetting what might otherwise have been improvements in health, perhaps even causing women who were affected by the program to have worse health in later life than women in the control area.

Does this mean that lower fertility and longer child spacing do not have the beneficial effects that have long been assumed? If the focus is on the later life health of the women who experience reduced fertility, then the Barham et al. results suggest that the answer may be yes. However, looking at the later-life health of the mothers is arguably not the place to look for the beneficial effects of family planning and lower fertility. The main beneficiaries of lower fertility are likely to be the children themselves. As shown in previous work by Barham (9), the children who were born to the women in the treatment area have a number of advantageous outcomes, including more schooling and higher cognitive performance, compared to children born in the comparison area. At ages 8 to 14, children born in the treatment area had 0.39 SD higher cognitive function than children born in the comparison area. For children who received for the full set of child health and nutrition interventions provided by the MCH/FP, the effect was almost twice as large (9). At ages 8 to 14, the children in the treatment area had 0.17 SD higher educational attainment and 0.22 SD higher height-for-age relative to children in the control area.

It is possible that the increased time and resources available to women when they have fewer children are mostly invested directly into their children. This is why children’s health and education increase rapidly as fertility declines. It is almost universally observed that when fertility rates decline, children’s health and education increase, a transition that I have argued is one of the fundamental components of economic development (2). This is consistent with the impact of women’s education on fertility in Brazil (11). Although Brazilian women with 8 y of schooling had significantly fewer children than women with 0 or 4 y of schooling in the early years of Brazil’s fertility decline, they did not have higher labor force participation. They did, however, have much healthier and better educated children. It appears that the increased productivity associated with greater education and the increased time and resources associated with lower fertility were heavily invested in the children.

The Barham et al. study is an important piece of evidence about the long-run impact of family planning programs. There are unlikely to be many other cases in which rich longitudinal data are available to track the outcomes of women affected by such an ambitious combination of programs targeted at maternal and child health and family planning. The fact that the long-term health of these women appears to be moderately worse than the health of women in the control group, despite the fact that the treated women had lower fertility and longer child spacing, will undoubtedly come as a surprise and disappointment to many of those involved in providing and studying family planning programs. However, it is important to keep in mind that these women were not the only beneficiaries of Matlab’s MCH/FP. There is good evidence that the children of these women benefited significantly in dimensions such as health, education, and cognitive ability. The mothers themselves may have played a role in diverting the additional time and resources they gained from having fewer children into improved outcomes for their children. Hopefully, future research will continue to follow these women, their children, and their children’s children to provide even greater insights into the long-term multigenerational impact of family planning programs.
